# Biosynthesis of ethylene glycol from d-xylose in recombinant *Escherichia coli*

**DOI:** 10.1080/21655979.2018.1478489

**Published:** 2018-07-03

**Authors:** Yuhui Wang, Mo Xian, Xinjun Feng, Min Liu, Guang Zhao

**Affiliations:** a CAS Key Laboratory of Biobased Materials, Qingdao Institute of Bioenergy and Bioprocess Technology, Chinese Academy of Sciences, Qingdao, China; b School of Life Science, Shandong University, Jinan, China; c Shandong Provincial Key Laboratory of Synthetic Biology, Qingdao, China

**Keywords:** Acetate, aldehyde reductase, biosynthesis, d-xylose, *Escherichia coli*, ethylene glycol, glycolate

## Abstract

Ethylene glycol (EG) is an important chemical used as antifreeze and a raw material in polyester synthesis. The EG biosynthetic pathway from D-xylose with D-xylonate as key intermediate has some advantages, but showed low EG production. Here, we reconstructed and optimized this pathway in *Escherichia coli*. In view of the greater intracellular prevalence of NADH, an aldehyde reductase FucO using NADH was employed to convert glycoaldehyde into EG, in replacement of NADPH-dependent reductase YqhD. To suppress the accumulation of by-products acetate and glycolate, two genes *arcA* and *aldA* were knocked out. The resultant strain Q2843 produced 72 g/L EG under fed-batch fermentation conditions, with the yield of 0.40 g/g D-xylose and EG productivity of 1.38 g/L/h. The use of NADH-dependent enzyme FucO and by-product elimination significantly improved the performance of EG producing strain, which represented the highest titer, yield and productivity of EG reported so far.

## Introduction

Ethylene glycol (EG) is an important platform chemical with considerable commercial value[1]. Almost all countries have used EG as an antifreeze since the 1950s. Furthermore, polyester products containing EG, including fiber, surfactants and plastics, have also been applied in many fields such as the textile industry and the catering industry[2]. The global demands for EG had reached 23.6 million tons in 2014 and its consumption is expected to increase[3]. At present, most of the chemicals derived primarily from fossil fuels, including EG, are non-renewable and non-sustainable [4,5]. Due to the depletion of fossil resources, growing attention has been paid to biosynthesis of value-added chemicals using renewable biomass[6].

In recent years, much effort has been made to increase bulk chemicals and biomaterials through metabolic engineering of microorganisms and synthetic biology methods [3,7]. Utilizing plant-derived substrates as feedstocks, such as sugar, starch and lignocellulose, to produce EG has been reported [8–10]. d-glucose and d-xylose are the two main constituents of lignocellulose. d-glucose can be easily metabolized by most microorganisms, so it was widely used for producing value-added chemicals [11,12]. d-xylose is the second most abundant sugar in nature, accounting for 18%-30% of sugar components in lignocelluloses[13]. However, only a small fraction of microorganisms can metabolize d-xylose. Therefore, it is worth exploring new pathways that convert d-xylose to chemicals such as butanetriol and glycolic acid.

**Figure 1.**
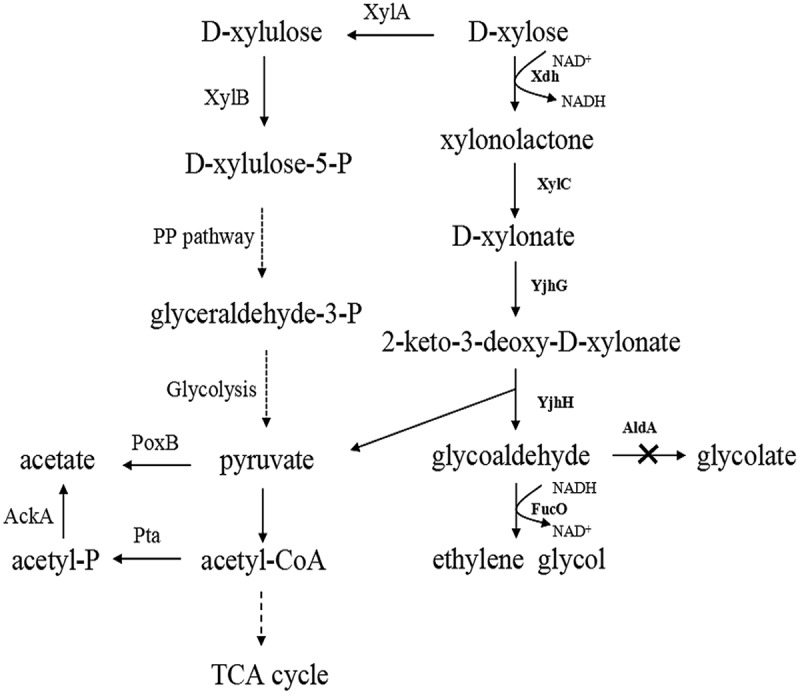
The metabolic pathway for production of ethylene glycol from d-xylose. Enzymes associated with the reactions were shown. d-xylose was converted into d-xylonate by xylose dehydrogenase Xdh and xylonolactonase XylC from *C. crescentus*. All other enzymes were from *E.coli* BL21 (DE3).

*Escherichia coli* can metabolize d-xylose through pentose phosphate pathway (Figure 1), but lacks the pathway for EG synthesis. Recently, three biosynthetic pathways of EG from d-xylose have been constructed in *E. coli*. The pathway with d-xylonate as key intermediate consists of four steps:

d-xylose → d-xylonate → 2-keto-3-deoxy-d-xylonate → glycoaldehyde → EG[14].

The other two pathways employed pentose isomerase and kinase to convert d-xylose into d-xylulose-1-phosphate or d-ribulose-1-phosphate, which was cleaved into glycoaldehyde and dihydroxyacetone phosphate [15,16]. Comparatively speaking, the pathway with d-xylonate as intermediate has some expected advantages, such as redox neutrality. The conversion of D-xylose into xylonolactone catalyzed by Xdh could produce one NADH in our constructed pathway. Meanwhile, the reaction catalyzed by the FucO consumed just one NADH. So the designed pathway is redox neutrality. Production of each mole of EG via the d-xylulose-1-phosphate pathway consumes one mole of ATP and one mole of NADH (Figure 1), whereas there is no phosphorylation step in our designed pathway. However, the engineered *E. coli* strain carrying the pathway with d-xylonate only produced 11.7 g/L EG after 48 h of fermentation, with the yield of 0.29 g/g D-xylose and productivity of 0.24 g/L/h[14], which cannot satisfy the needs of large scale industrial production [17,18]. Further development to achieve a high EG yield via this pathway could be carried out in two aspects. First, the glycoaldehyde reductase YqhD used in the EG biosynthetic pathway prefers NADPH as coenzyme, while the intracellular concentration of NADPH is much lower than that of NADH[19]. Studies investigating glycoaldehyde reductase using NADH as coenzyme could solve this problem. Secondly, suppressing the production of competitive byproduct glycolate and acetate may also enhance the production of EG.

In this study, we have optimized the d-xylonate pathway for EG biosynthesis and achieved the greatest amount of EG reported so far. In detail, the reductase FucO using NADH as coenzyme was employed to convert glycoaldehyde into EG. To eliminate the production of glycolate, *aldA* gene encoding aldehyde dehydrogenase was knocked out. To suppress the production of acetate, genes encoding ArcA (global transcriptional regulator) were deleted.

## Results and discussion

### Construction and assessment of the EG biosynthetic pathway

Five enzymes are involved in the EG biosynthesis via the d-xylonate pathway (Figure 1). d-xylose dehydrogenase (Xdh) and xylonolactonase (XylC) were recruited from *Caulobacter crescentus*, and cooperatively converted d-xylose into d-xylonate[20]. which was further transformed into 2-keto-3-deoxy – d-xylonate by the endogenous D-xylonate dehydratase YjhG. Split of 2-keto-3-deoxy- d-xylonate into glycoaldehyde and pyruvate was catalyzed by the endogenous aldolase YjhH, and the reduction of glycoaldehyde to EG was accomplished by the aldehyde reductase FucO using NADH as coenzyme[15]. All these genes were cloned and overexpressed in *E. coli* BL21 (DE3), generating the engineered strain Q2766. Considering that NADH is more prevalent than NADPH in the cell, it is expected that this pathway will increase EG production to levels better than pathways employing aldehyde reductase YqhD, which requires NADPH as coenzyme[15].

**Figure 2.**
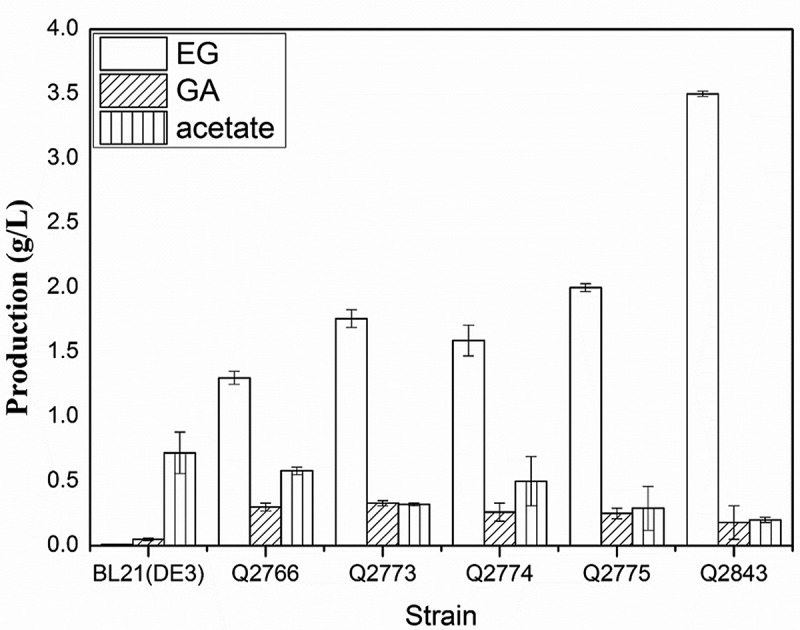
Concentration of the ethylene glycol, glycolate and acetate produced by the strains *E. coli* BL21 (DE3), Q2766, Q2773, Q2774, Q2775, and Q2843 in the shaking flask cultivation. Columns were the average of triplicate experiments, and errors bars represent standard deviation.

**Figure 3.**
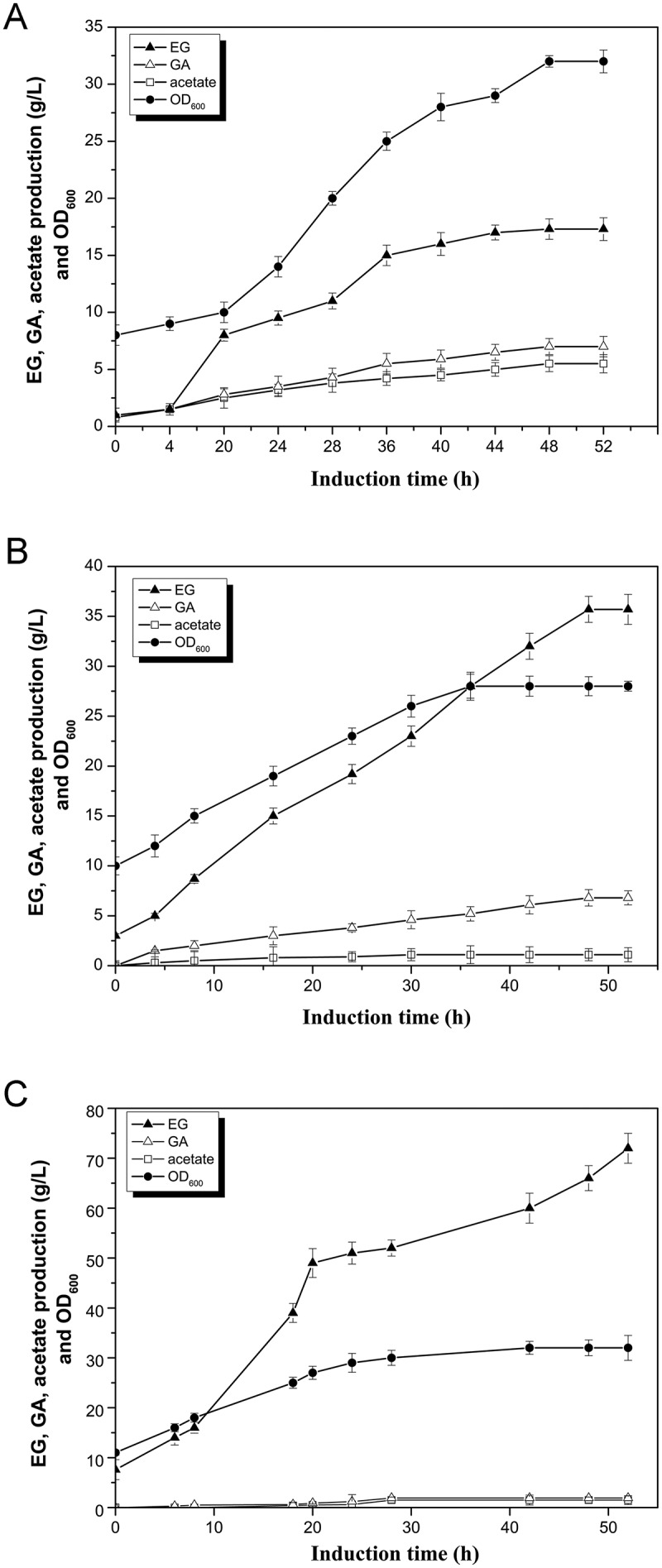
Time profiles for the OD_600_ and production of ethylene glycol, glycolate and acetate of the strain Q2766 (A), Q2775 (B), and Q2843 (C) under fed-batch fermentation. EG, ethylene glycol; GA, glycolate.

For EG production, control strain *E. coli* BL21 (DE3) carrying empty vectors and Q2766 were cultivated with d-xylose as the sole carbon source. After 48h cultivation, 1.30 ± 0.05 g/L EG was accumulated in Q2766, whereas it was not detected in *E. coli* BL21 (DE3) culture (Figure 2). Furthermore, laboratory-scale fermentation with the engineered strain Q2766 was performed in a 3 L bioreactor under fed-batch model. The EG production of the engineered strain Q2766 was 17.3 g/L, with the yield of 0.17 g/g D-xylose and the productivity of 0.33 g/L/h (Figure 3A). Compared to the results of Liu et al. (2013), both EG titer and productivity have been improved significantly, indicating the important role of NADP-dependent reductase FucO in EG production.

The natural D-xylose metabolic pathway of *E. coli* termed XI pathway was described in Figure 1. The XI pathway can convert D-xylose into D-xylose-5-P via the isomerization and phosphorylation which was catalyzed by the XylA and XylB, and then D-xylose-5-P entered the pentose phosphate pathway to participate in central metabolism. In our designed pathway, the Xdh and XylC of *C. crescentus* were introduced to metabolize xylose to D-xylonate. Then D-xylonate was split into pyruvate via the dehydration and entered the EMP directly. Compared with the *E. coli* natural XI pathway, the synthetic pathway shortened the xylose assimilation process which may improve the efficiency of the pathway theoretically.

In both shaking flask cultivation and fed-batch fermentation progress, the undesirable by-products such as acetate and glycolate were also detected (Figure 2 and Figure 3A). Acetate excretion affects the cell growth and accumulation of desirable product even at concentration as low as 0.5 g/L[21]. Glycolate is a carbon-competing by product during EG production as both glycolate and EG are derived from the same precursor glycoaldehyde (Figure 1). Regulation strategies were carried out to suppress the accumulations of acetate and glycolate and increase the EG production.

### Overcoming acetate overflow

The pathways for acetate formation in *E. coli* were shown in Figure 1. Three genes encoding regulators or enzymes related to acetate metabolism were knocked out to test their effect on suppressing acetate accumulation, respectively. The *arcA* gene encodes the regulator of ArcAB two-component system, which controls expression of numerous genes of the TCA cycle and the glyoxylate shunt[22], and it was reported that the *arcA* mutant strain undergoes a significant reduction in acetate accumulation and improvement in target production [23,24]. The *iclR* gene encodes isocitrate lysate regulator that suppresses the expression of the *aceBAK* operon in the glyoxylate shunt [25,26]. The glyoxylate shunt is the main pathway for acetate utilization, and deletion of *iclR* gene will relieve the inhibitory effect of *aceBAK* operon and elevate the assimilation of acetate. The *ackA* gene encodes acetate kinase, which directly produces acetate from acetyl phosphate.

Following shaking flask cultivation, all three mutations increased the EG titer and suppressed acetate concentration, and the *arcA* mutant Q2775 achieved the highest EG production of 2.0 ± 0.03 g/L (Figure 2). Therefore, strain Q2775 was further tested under fed-batch fermentation conditions. As expected, the acetate concentration was controlled at a reasonable level of 0.8 g/L, and EG was accumulated to 35.7 g/L (Figure 3B), 2.06-time higher than that of strain Q2766. ArcA is a global regulator that usually controls several operons belonging to different groups and exhibits pleiotropic phenotypes. Knockout of *arcA* can enhance the activity of the TCA cycle and the glyoxylate shunt and elevate acetate assimilation. Presumably, metabolic engineering of *arcA* can redistribute the carbon flux globally to overcome undesirable consumption and improve EG production. Due to these advantages, the global transcriptional regulators have been widely used for improving target production in recent years.

### Suppressing glycolate production

Although deletion of *arcA* gene significantly increased the EG production and decreased acetate excretion, another by-product glycolate still accumulated during strain Q2775 fermentation (Figure 3B). Glycolate is formed from the oxidation of glycolaldehyde catalyzed by aldehyde dehydrogenase (encoded by *aldA*) (Figure 1). Therefore we tested the effect of deleting this gene on EG production.

An *arcA aldA* double mutant strain Q2843 was constructed and cultivated under shaking flask and fed-batch fermentation conditions. In shaking flask, the strain Q2843 produced 3.52 ± 0.13 g/L EG, and the concentration of glycolate was 0.18 ± 0.13 g/L (Figure 2). In bioreactor, the strain Q2843 accumulated 72 g/L EG and 1.5 g/L glycolate after 52 h fermentation (Figure 3C). Comparison of the shake flask and bioreactor-fermentation indicates that under controlled oxygen supply the effect of the *aldA* deletion is much more prominent. Overall, our results indicated that deletion of *aldA* gene largely contributed to improving EG biosynthesis. It is interesting to notice that the observed positive effect of *aldA* mutation agrees with some previous reports, [3,10] and disagrees with data of Liu et al. (2013) who found that the *aldA* deletion was toxic for the cells.

Some studies of EG biosynthesis from d-xylose were summarized in Table 3. The pathway with d-xylonate as key intermediate has some expected advantages as it does not require reducing power and energy, and has been implicated in *E. coli* and *Saccharomyces cerevisiae*[27]. There were two major differences between our work and previous studies: use of a NADH-dependent aldehyde reductase and suppression of acetate and glycolate production, which significantly increased the EG yield and titer. Our recombinant *E. coli* strain accumulated 72 g/L EG, with the yield of 0.40 g/g d-xylose and the productivity of 1.38 g/L/h. To the best of our knowledge, this is the highest EG production.

**Table 1.** Primers used in this study.

Name	Sequence
*yjhH*_F_*Nco*I	GGAATTCCATATGAAAAAATTCAGCGGCATTATTC
*yjhH*_R_*Eco*RI	CCGCTCGAGTCAGACTGGTAAAATGCCCT
*xylC*_F_ *Eco*RI	CCGGAATTCTAATACGACTCACTATAGGGGAATTG
*xylC*_R_*Not*I	AAGGAAAAAAGCGGCCGCTTAAACCAGACGAACTTCGTGCTG
*fucO*_F_ *Nco*I	CCGCCATGGGCATGGCTAACAGAATGATTCTG
*fucO*_R_ *Eco*RI	CCGGAATTCTTACCAGGCGGTATGGTAAAG
*xdh*_F_*Nde*I	GGGAATTCCATATG TCCTCAGCCATCTATCCC
*xdh*_R_*Kpn*I	CGGGGTACC TCAACGCCAGCCGGCGTCGAT
*yjhG*_F_*Nde*I	GGAATTCCATATG TCTGTTCGCAATATTTTTGC
*yjhG*_R_*XhoI*	CCGCTCGAGTCAGTTTTTATTCATAAAATCGCG
*aldA*_UP_F	TTATCCCGGATTCGTTCCTC
*aldA*_UP_R	GCTTCCCATTCTGGTTGTGC
*aldA*_DOWN_F	GCACAACCAGAATGGGAAGC GCTATGCAAGGCTTCCACGC
*aldA*_DOWN_R	GCATGACAGTTTCGGGATAG
ID- *aldA* _F	GATATCGCTCATCAGTTCTG
ID- *aldA* _R	CGTCAATGACGACACTCTGG
ID-*arcA*_F	GTTATCAACAAGTTATCAAGT
ID-*iclR*_F	CATAAAACGGATCGCATAACGC
ID-*ackA*_F	CATAAAACGGATCGCATAACGC

**Table 2.** Plasmids and strains used in this study.

Plasmids and strains		
Plasmids	Description	Source
pETDuet1	*Amp^r^ oriP_BR322_ lacI^q^ P_T7_*	Novagen
pACYCDuet1	*Cm^r^ oriP_15A_ lacI^q^ P_T7_*	Novagen
pETDuet1-*yjhH-xylC-xdh*	*Amp^r^ oriP_BR322_ lacI^q^ P_T7_ yjhH P_T7_xylC P_T7_xdh*	This study
pACYCDuet1-*fucO-yjhG*	*Cm^r^ oriP_15A_ lacI^q^ P_T7_ fucO P_T7_yjhG*	This study
**Strains**		
*E. coli* DH5α	F^−^ *supE*44 Δ*lacU*169 (*φ*80 *lacZ* φ*M15*) *hsdR*17 *recA*1 *endA*1 *gyrA*96 *thi*-1 *relA*1	Invitrogen
*E. coli* BL21(DE3)	F^−^ *ompT gal dcm lon hsdSB* (rB^−^ mB^−^) λ(DE3)	Invitrogen
Q1949	*E. coli* BL21(DE3)△*arcA*	This study
Q1951	*E. coli* BL21(DE3)△*ackA*	This study
Q2280	*E. coli* BL21(DE3)△*iclR*	This study
Q2766	*E. coli* BL21(DE3)*/*pETDuet1*-yjhH-xdh-xylC/* pACYCDuet1*-fucO-yjhG*	This study
Q2773	*E. coli* BL21(DE3) △*ackA*/pETDuet1-*yjhH-xdh-xylC*/ pACYCDuet1-*fucO-yjhG*	This study
Q2774	*E. coli* BL21(DE3)△*iclR*/pETDuet1-*yjhH-xdh-xylC*/ pACYCDuet1*-fucO-yjhG*	This study
Q2775	*E. coli* BL21(DE3)△*arcA/*pETDuet1-*yjhH-xdh-xylC*/ pACYCDuet1-*fucO-yjhG*	This study
Q2827	*E. coli* BL21(DE3)△*arcA*△*aldA*	This study
Q2843	*E. coli* BL21(DE3)△*arcA*△*aldA/* pETDuet1-*yjhH-xdh-xylC*/pACYCDuet1-*fucO-yjhG*	This study

**Table 3.** Comparison of EG production from d-xylose by different strains.

Strain	Pathway	Coenzyme required	NAD(P)H yield (mole/mole EG)	ATP yield (mole/mole EG)	EG production (g/L)	Yield (g/g d-xylose)	Productivity (g/L/h)	References
*E. coli*	d-xyl → XA → KDXA → GAH → EG	NADH	0	0	72	0.40	1.38	This study
*E. coli*	d-xyl → XA → KDXA → GAH → EG	NADPH	0	0	11.7	0.29	0.24	Liu et al. 2013
*E. coli*	d-xyl → XA → KDXA → GAH → EG	NADPH	0	0	7.72	0.39	0.06	Cabulong et al. 2017
*S. cerevisiae*	d-xyl → XA → KDXA → GAH → EG	NA	0	0	0.014	-	-	Salusjarvi et al. 2017
*E. coli*	d-xyl → d-rib → d-rib-1P → GAH → EG	NADH	−1	−1	40	0.35	0.56	Pereira et al. 2016a
*E. coli*	d-xyl → d-xylu → d-xylu-1P → GAH → EG	NADH	−1	−1	20	0.38	0.37	Alkim et al. 2015

d-xyl, d-xylose; XA, d-xylonate; KDXA, 2-keto-3-deoxy-d-xylonate; GAH, glycoaldehyde; d-rib, d-ribulose; d-rib-1P, d-ribulose-1-phosphate; d-xylu, d-xylulose; d-xylu-1P, d-xylulose-1-phosphate.

## Materials and methods

### The construction of recombinant plasmids

The primers used in this study are shown in Table 1, and a series of the bacterial strains and plasmids used in this work are shown in Table 2. *E. coli* BL21 (DE3) was selected as the host bacteria and DH5α served as the cloning host. *E. coli* CC118 and the diaminopimelic acid (DAP) auxotroph bacteria *E. coli* χ7213 were in collaboration, with pRE112 mediating homologous recombination for genetic modifications. Ampicillin (100 mg/L) and chloramphenicol (34 mg/L) were supplemented when necessary.

The d-xylose dehydrogenase gene *xdh* (Acession number: MG189911) and the xylonolactonase gene *xylC* (Acession number: MG189912) that were codon-optimized for *E. coli* were synthesized from the Beijing Genomics Institution, and cloned into the *Nde*I/*Kpn*I and *Eco*RI/*Not*I sites of vector pETDuet-1 to generate plasmid pETDuet1-*xdh-xylC*. The gene *yjhH* (GeneID: 948825) was amplified from the *E. coli* genome and inserted into *Nco*I/*Eco*RI site of pETDuet1*-xdh-xylC*. The genes of *fucO* (GeneID:947273) and *yjhG* (GeneID:946829) were cloned into pACYCDute1between the *Nco*I/*Eco*RI and *Nde*I/*Xho*I sites, resulting in pACYCDute1-*fucO-yjhG*.

### The construction of mutant strains

All the *E. coli* single-gene-knockout mutants were constructed by allelic exchange using the suicide vector pRE112[28]. For example, the upstream and downstream homologous fragments of *arcA* were amplified from the *E. coli* genome, and ligated by overlapping PCR. The PCR product was cloned into the *Kpn*I/*Sac*I site of pRE112 vector to generate plasmid pRE112-Δ*arcA*. The *arcA* mutant was gained by allelic exchange using pRE112-Δ*arcA*, and named Q1949. Diaminopimelic acid (50 mg/mL) was used for the growth of *E. coli* χ7213 strain and 10% sucrose was added to LB plates as a selection marker in the allelic exchange experiments.

### Shaking flask cultivation

Shaking flask cultivation was used to evaluate the ability to produce EG in different engineered strains. An optimized minimal medium (MM) was used, comprising 14 g/L dipotassium hydrogen phosphate, 5.2 g/L potassium dihydrogen phosphate, 1 g/L sodium chloride, 1 g/L ammonium chloride, 0.25 g/L magnesium sulfate heptahydrate, 0.2 g/L yeast extract, and 15 g/L d-xylose. 10 M sodium hydroxide was used to adjust the pH to approximately 7.0. The shaking flask fermentation was carried out in triplicate in a 250 mL shake flask containing 50 mL MM. All engineered strains were grown overnight in 3 mL LB broth, diluted 1:50 into 50 mL MM, and further cultivated with shaking at 180 rpm at 37ºC. Then, 0.1 mM IPTG was added, and the temperature was reduced to 30° when the OD_600_ reached 0.6 – 0.8.

### Fed-batch fermentation

The engineered strains were grown in Biostat B plus MO3L fermenters (Sartorius, Germany) in 1 L medium. The fermentation medium was composed of 9.8 g/L dipotassium hydrogen phosphate, 2.1 g/L citric acid•H_2_O, 0.3 g/L ferric ammonium citrate, 3.0 g/L ammonium sulfate, 0.2 g/L magnesium sulfate heptahydrate, 20 g/L d-xylose, 3 g/L yeast extract, and 1000 × trace metal (3.7 g/L ammonium molybdate, 2.9 g/L zincsulfate, 24.7 g/L orthoboric acid, 2.5 g/L copper(II) sulfate pentahydrate, and 15.8 g/L manganese(II) chloride). d-xylose was the sole carbon source during the entire fermentation. The 100 mL seed cultures were grown in fermentation medium at 37°C at 180 rpm and transferred into the bioreactor. Ammonia and 10% H_2_SO_4_ were used to maintain the pH at 7.0 automatically. The temperature was set to 37°C, and 30% dissolved oxygen content was preserved via regulating the agitation from 300 to 800 rpm. After the 15 g/L initial D-xylose was nearly depleted, 60% (W/V) D-xylose was fed into the bioreactor. In the process of fermentation, the residual D-xylose was maintained below 5 g/L. The supply rate was adjusted depending on the concentration of residual D-xylose in the bioreactor which detected every 2 h. When the OD_600_ reached 10 ~ 15, the temperature was adjusted to 30° and 0.1 mM IPTG was added to the cell culture.

## Analytical methods

The biomass was assayed via measuring the optical density at 600 nm (Varian Cary 50 Bio, US). The cells were precipitated by centrifugation, and the supernatant was analyzed by the Agilent 1200 HPLC system, coupled with an AminexHPX-87H column (Bio-Rad, Hercules, CA) and a differential refractive index detector. The column was heated to 50°, and the mobile phase of 0.5 mM H_2_SO_4_ was run at 0.5 mL/min.

## Conclusion

EG is an important platform chemical that is widely used in many different fields. It is promising to produce EG from d-xylose, the second most abundant sugar in nature. In this work, we focused on reconstruction and optimization of the EG biosynthetic pathway from d-xylose with d-xylonate as key intermediate. Replacement of NADPH-dependent aldehyde reductase YqhD by FucO using NADH and deletion of *arcA* and *aldA* genes both largely contributed to the increased EG production. Finally, under fed-batch conditions, our recombinant strain Q2843 gained the highest titer, yield and productivity of EG reported so far. This study demonstrates potential for microbial production of EG from renewable resources.
